# The Astrocytic cAMP Pathway in Health and Disease

**DOI:** 10.3390/ijms20030779

**Published:** 2019-02-12

**Authors:** Zhiwen Zhou, Yuji Ikegaya, Ryuta Koyama

**Affiliations:** 1 Laboratory of Chemical Pharmacology, Graduate School of Pharmaceutical Sciences, The University of Tokyo, 7-3-1 Hongo Bunkyo-ku, Tokyo 113-0033, Japan; zhouzhiwen.star@gmail.com (Z.Z.), yuji@ikegaya.jp (Y.I.); 2 Center for Information and Neural Networks, Suita City, Osaka 565-0871, Japan

**Keywords:** astrocyte, cAMP, adenylyl cyclase, lactate shuttle, glycogenolysis

## Abstract

Astrocytes are major glial cells that play critical roles in brain homeostasis. Abnormalities in astrocytic functions can lead to brain disorders. Astrocytes also respond to injury and disease through gliosis and immune activation, which can be both protective and detrimental. Thus, it is essential to elucidate the function of astrocytes in order to understand the physiology of the brain to develop therapeutic strategies against brain diseases. Cyclic adenosine monophosphate (cAMP) is a major second messenger that triggers various downstream cellular machinery in a wide variety of cells. The functions of astrocytes have also been suggested as being regulated by cAMP. Here, we summarize the possible roles of cAMP signaling in regulating the functions of astrocytes. Specifically, we introduce the ways in which cAMP pathways are involved in astrocyte functions, including (1) energy supply, (2) maintenance of the extracellular environment, (3) immune response, and (4) a potential role as a provider of trophic factors, and we discuss how these cAMP-regulated processes can affect brain functions in health and disease.

## 1. Introduction

Astrocytes are a major class of glial cells that play a critical role in maintaining the homeostasis of the central nervous system (CNS). Astrocytes occupy the overwhelming majority of space in the brain and interact with synapses and blood vessels as well as with other glial cells via their intricate and fine processes. Thus, astrocytes are in an ideal position to store and supply energy, maintain the extracellular environment after neuronal activity, supply trophic factors, and engage the immune response by interacting with microglia and peripheral immune cells [1]. These functions are important for maintaining proper neuronal activity, preventing oxidative stress, and enabling basic brain functions such as learning and memory [2,3,4,5]. Breakdown of the supportive functions and immune reactivity of astrocytes is associated with numerous neural diseases, including epilepsy, depression, multiple sclerosis, brain edema, and Alzheimer’s disease [6,7,8,9,10].

Astrocytes have also been suggested as playing important roles in cognitive functions. Species with larger brains and higher cognitive capabilities have larger numbers of astrocytes and higher astrocyte-to-neuron ratios [1]. Recently, several studies have shown that astrocytes act as a component of neural circuits by transducing signals to neurons through the release of gliotransmitters [11,12,13,14,15]. The fine tuning of neuronal activity by gliotransmitters may lead to the refinement of cognitive capabilities.

Diverse functions of astrocytes are partly regulated by cyclic adenosine monophosphate (cAMP) signaling. cAMP has been shown to trigger glycogenolysis-related energy supply and regulate homeostasis-sustaining functions such as glutamate uptake, potassium buffering, and water permeability. Additionally, cAMP is a major player in astrocytic immune response, in which it regulates cytokine and inflammatory factor release [16]. cAMP also has a complex relationship with Ca^2+^, another important second messenger that has been extensively studied in astrocytes. cAMP has been shown to regulate the Ca^2+^ current of voltage-dependent L-type calcium channels [17,18]. Recently, cAMP elevation in acute hippocampal slices has been shown to elicit oscillating Ca^2+^ activity in astrocytes [19]. Increases in Ca^2+^ have been linked to the release of gliotransmitters, including adenosine triphosphate (ATP), glutamate, and d-serine, which can adjust neuronal activity and affect synaptic plasticity [20,21]. Astrocytes express various G protein-coupled proteins (GPCRs) that can be activated by neurotransmitters, neuromodulators, and neuropeptides to up- or down-regulate cAMP levels in cells [16]. Thus, the cAMP level in astrocytes is considered to be constantly and dynamically regulated by the extracellular environment.

In this review, we will introduce the role of astrocytic cAMP in (1) energy supply, (2) maintenance of the extracellular environment, (3) immune response, and (4) a potential role as a provider of trophic factors, and we will discuss how these cAMP-regulated processes can affect the healthy and diseased CNS.

## 2. The cAMP Pathway in Astrocytes

### 2.1. cAMP Synthesis in Astrocytes

cAMP is synthesized from ATP by adenylyl cyclase (AC). Astrocytes express various GPCRs that can be activated by neurotransmitters, neuromodulators, neuropeptides, and hormones, and some GPCRs are coupled to transmembrane AC to produce cAMP.

One major source of astrocytic cAMP is the activation of β-adrenergic receptors. Astrocytes express all three types of β-adrenergic receptors (β_1_, β_2,_ and β_3_), which can elevate intracellular cAMP levels upon activation [22,23,24]. In vivo Ca^2+^ imaging has revealed that Ca^2+^ activity in cortical astrocytes is mainly triggered by noradrenaline, suggesting the possibility that astrocytic cAMP may also be elevated by noradrenaline through activation of β-receptors [25]. Knockdown of astrocytic β_2_ receptors in the hippocampus results in the impairment of memory consolidation, which implies that astrocytic cAMP levels are also increased by β-receptors during memory processes [26].

Astrocytes also express all four types of adenosine receptors. The A2A and A2B adenosine receptors are coupled to AC, and the A2B receptor has been shown to be highly expressed in cortical astrocytes [27]. Because adenosine accumulates during wakefulness, extracellular adenosine can constantly activate astrocytic adenosine receptors to create a basal level of cAMP tone [28]. Indeed, the blockade of transmembrane AC in cultured astrocytes results in a decrease in intracellular cAMP levels [29].

Other neuromodulators, including serotonin, dopamine, and histamine, can also activate AC-coupled GPCRs on astrocytes. The 5-HT_5A_ receptor, which can activate AC, is expressed by astrocytes [27], and 5-HT_4_ receptor activation has been shown to regulate astrocytic immune response [30]. However, it should be noted that 5-HT_5A_ and 5-HT_4_ receptors may not be the predominant serotonin receptors in astrocytes [31]. Astrocytic expression of dopamine receptors is mainly found in the striatum. AC-activating D1 and D5 receptors are expressed in astrocytes [32]. Interestingly, dopamine has also been found to activate β-adrenergic receptors in cultured astrocytes [33]. An autoradiographic study has shown that astrocytes express histamine H1 and H2 receptors and that the histamine-induced cAMP increase in astrocytes is mainly mediated by H2 receptors [34,35].

Neuropeptides such as vasoactive intestinal peptide (VIP) and pituitary adenylyl cyclase activating polypeptide (PACAP) can activate AC upon binding to VPAC1, VPAC2, and PAC1 receptors, which are expressed by astrocytes throughout the brain [36,37].

In addition to transmembrane AC, which is usually coupled to GPCRs, astrocytes also express soluble AC (sAC). sAC was first found in testis tissue and can be activated by HCO_3_^−^ to promote sperm motility [38]. In astrocytes, it has been shown that an increase in extracellular K^+^ can activate the electrogenic NaHCO_3_ cotransporter to import HCO_3_^−^, which then activates sAC to produce cAMP [39]. Given that K^+^ is constantly released into the extracellular space via neuronal activity, astrocyte sAC can be activated along with changes in neuronal activity. Extracellular K^+^ concentration has been shown to be elevated during slow-wave sleep, especially after seizure attacks, potentially activating sAC and increasing astrocytic cAMP [40].

Other stimuli such as the binding of astrocytic intercellular adhesion molecule 1 (ICAM-1) can also cause intracellular cAMP accumulation in astrocytes [41]. In addition, lactate, fatty acids, aspirin, and numerous antidepressants have been shown to increase astrocytic cAMP; however, the underlying mechanisms remain largely unclear [42,43,44].

### 2.2. Pathways Downstream of Astrocytic cAMP

cAMP in astrocytes transduces signals via protein kinase A (PKA), Epac1 (exchange factor directly activated by cAMP 1) and Epac2 as well as hyperpolarization-activated cyclic nucleotide–gated (HCN) channels. RNA sequencing analysis suggests the likelihood that PKA and Epac1 are the predominantly expressed proteins downstream of cAMP in astrocytes [45,46]. It has also been shown that HCN channels are expressed in astrocytes after ischemia [47]. The activation of Epac2 in neural precursors by PACAP has been shown to facilitate astrocytic differentiation [44,48]. After being synthesized, cAMP is rapidly degraded into AMP by phosphodiesterases (PDEs), which also degrade cyclic guanosine monophosphate (cGMP). Astrocytes mainly express PDE1 and PDE4 (cAMP specific), with markedly elevated expression of PDE4B in cortical astrocytes [45,46]. Astrocytic PDE4 expression can also be upregulated by cytokines [49].

cAMP elevation can also increase intracellular AMP, which then activates AMP-activated protein kinase (AMPK), although this process is not considered to be the canonical intracellular cAMP pathway. Astrocytic AMPK has been shown to be important in glutamate metabolism [50].

Leakage of cAMP into the extracellular space was first reported some decades ago. β-Adrenergic receptor activation causes an increase in extracellular cAMP in astrocytic cultures. Extracellular cAMP is then degraded into adenosine, which may activate the adenosine receptors of surrounding cells [51,52].

In conclusion, astrocytes possess the machinery to increase intracellular cAMP and contain downstream targets. Although direct evidence of cAMP elevation in astrocytes in vivo is still scarce, there is a reasonable possibility that astrocytic cAMP increases under multiple circumstances in vivo in response to various stimuli and that it regulates astrocytic function.

## 3. Glycogenolysis and Lactate Release from Astrocytes

### 3.1. Glycogenolysis in Astrocytes and Its Dependence on cAMP

One fundamental role of astrocytes is to provide energy substrates to other cells, especially neurons. Astrocytes are anatomically in contact with brain capillaries and neurons, which puts them in a perfect position for taking up glucose from blood vessels and supplying energy to neurons. Indeed, astrocytes have been shown to be the almost exclusive store of glycogen in the brain both decades ago by electron microscopy [53] and by immunohistochemistry in microwave-fixed mouse brains recently [54]. In addition, in vitro experiments have shown that the expression of proteins involved in astrocytic energy metabolism is induced when astrocytes are cocultured with activated neurons [55].

**Figure 1:**
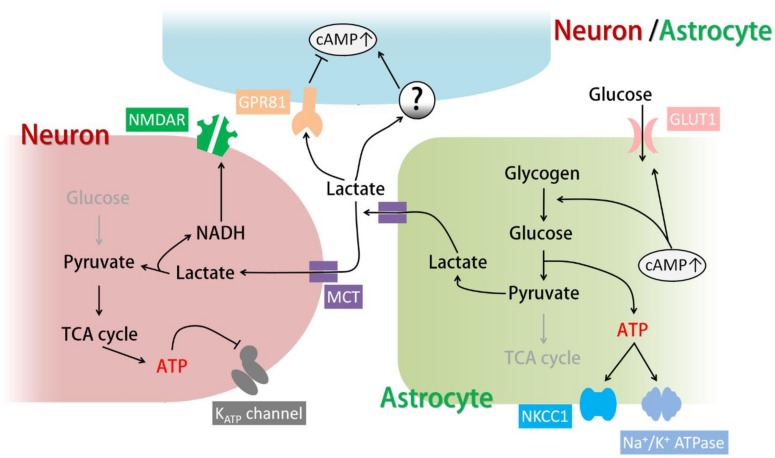
Energy metabolism and lactate shuttling between astrocytes and neurons. Astrocytic cAMP increases glucose uptake and glycogenolysis, triggering glycolysis and energy production. ATP produced from glycolysis is essential for K^+^ uptake by Na-K-Cl cotransporter 1 (NKCC1) and Na^+^/K^+^ ATPase. In the meantime, pyruvate in astrocytes is converted into lactate and shuttled to neurons via monocarboxylate transporters (MCTs). Lactate is converted into pyruvate for energy production in neurons and deactivates the K_ATP_ channel, resulting in an increase in neuronal excitability. The co-product NADH (reduced form of nicotinamide adenine dinucleotide) has been shown to be important for N-Methyl-D-aspartic acid receptor (NMDAR) activation, which is important for synaptic plasticity. In addition, extracellular lactate can activate G-protein-coupled receptor 81 (GPR81) and unknown targets to either up- or down-regulate cAMP levels in neurons and astrocytes. Grey arrows and texts indicate that the processes shown are less active. Arrows indicate transference or conversion of molecules, or activating pathways. T-bars indicate inhibiting pathways.

When in demand, glycogen is broken down by glycogen phosphorylases into phosphorylated glucose, a process known as glycogenolysis. Phosphorylated glucose is then utilized in energy metabolism. Glycogenolysis is triggered by an elevation of cytosolic cAMP and/or Ca^2+^ both in the brain and in the peripheral tissues. Numerous transmitters can cause glycogenolysis through AC-coupled GPCRs (Figure 1). For example, noradrenaline and isoproterenol have been shown to trigger glycogenolysis through β-adrenergic receptors; additionally, VIP acting via VPAC receptors and adenosine acting via A2A receptors can also activate glycogenolysis in cultured astrocytes [56,57]. Recently, it has been reported that fatty acids activate transmembrane AC and increase astrocytic lactate release in a cAMP-dependent manner [43]. In addition, increases in extracellular K^+^, which are well known to cause glycogenolysis, have also been shown to trigger glycogenolysis in acute rat brain slices through elevation of cAMP and consequent activation of sAC [39]. Notably, it has been argued that cAMP alone is not sufficient to induce glycogenolysis and is absolutely dependent on intracellular Ca^2+^ increases [57]. However, the cAMP analogue Dibutyryl-cAMP (dbcAMP) and the AC activator forskolin have been shown to trigger glycogenolysis in cultured astrocytes, and inhibitors of PKA and sAC have been shown to completely block astrocytic glycogenolysis induced by isoproterenol or extracellular K^+^ increases [39,57]. These findings suggest that cAMP can act as the dominant triggering signal for glycogenolysis. Other experiments on cultured mouse astrocytes show that treatment with a low concentration of a cAMP analogue or a protein kinase C (PKC) activator alone can induce a small degree of glycogenolysis, while the combination of both reagents greatly enhances glycogenolysis [56]. Given the complexity of GPCRs and the intricate relationship between cAMP and Ca^2+^ signals, it is plausible that a single transmitter or stimulus can raise intracellular cAMP and Ca^2+^ at the same time to activate glycogenolysis.

In addition to inducing glycogenolysis, cAMP signaling in astrocytes also regulates glucose uptake and glycogen synthesis (Figure 1). All three types of β-adrenergic receptors have been shown to cause glucose uptake upon activation in mouse astrocytes [23]. β_2_ receptors have been shown to promote glucose uptake through glucose transporter 1 (GLUT1) [58]. In chicken astrocytes, β_2_ and β_3_ receptors are responsible for glucose uptake [59]. On the other hand, adenosine has been shown to increase glycogen content in astrocytes in the long term via the A2B receptor-PKA pathway (Figure 1). A2B receptor activation increases the expression of protein targeting to glycogen (PTG), which promotes glycogen synthesis [60].

### 3.2. Functions of Astrocytic Glycogenolysis and the Lactate Shuttle in Relation to cAMP Signals

Glycogenolysis is essential in supplying energy for astrocytic functions that enable brain homeostasis, including K^+^ uptake through the Na^+^/K^+^ ATPase (Figure 1) [61], glutamate uptake and recycling [5], and release of ATP as a gliotransmitter. When some of these processes are triggered, glycogenolysis is subsequently activated [57,62]. Glycogen levels have been shown to decrease after memory tasks and even sensory input, probably because maintenance of the extracellular environment is in demand, especially after increased neuronal activity [63,64].

Interestingly, astrocytic glycogenolysis is also important for neuronal activity itself and the subsequent plastic changes [3]. It has been well documented that inhibition of glycogenolysis can result in failure of neuronal functions, especially in learning and memory. Object recognition learning in young chickens is reportedly disrupted upon glycogenolysis inhibition, as are passive avoidance learning, cocaine-induced conditioned place preference, and spatial working memory in rats [3,65,66,67]. Given the high level of energy consumption required for neuronal activity, glycogenolysis inhibition attenuates the energy supply from astrocytes to neurons and causes disruptions in learning and memory [57].

The astrocytic energy supply to neurons consists of the well-known “astrocyte-neuron lactate shuttle (ANLS)”: pyruvate produced by glycogenolysis and glycolysis is converted into lactate by lactate dehydrogenase (LDH) in astrocytes and released through monocarboxylate transporter 1 (MCT1) and MCT4, which are expressed in astrocytes (MCT1 is expressed in other glia as well). Extracellular lactate is then taken up by neurons through MCT2 and converted again into pyruvate for energy metabolism (Figure 1) [3,68]. Since inhibition of the ANLS by MCT blockers disrupts learning and memory, while lactate rescues the memory impairment caused by glycogenolysis inhibition, ANLS is believed to be triggered by glycogenolysis and is considered essential for neuronal activity and plasticity [3,66,67]. This theory is consistent with the fact that energy metabolism in neurons is mainly oxidation while glycolysis is restricted, which suggests that lactate is a more suitable neuronal energy substrate than glucose [45]. Although astrocytes store glycogen and possess elevated glycolytic activity, the enzyme activity of pyruvate dehydrogenase (PDH) which processes pyruvate into acetyl coenzyme A for tricarboxylic acid (TCA) cycle is limited [69]. Thus, these systems together make lactate the most efficient energy supply from astrocytes to neurons (Figure 1). It should be noted that astrocytes specifically express pyruvate carboxylase which enters pyruvate into the TCA cycle as oxaloacetate [70]. However, this process is considered crucial for de novo synthesis of neurotransmitter glutamate and GABA rather than the production of energy [70,71].

The ANLS was originally proposed and shown to be activated by glutamate uptake through glutamate transporters, which can act as a sensor of neuronal activity [62]. More recently, it has been proposed that the ANLS can be triggered by VIP released from VIP-expressing neurons throughout the cortex and by noradrenaline released from noradrenergic terminals originating from neurons in the locus coeruleus [69]. Noradrenaline and its Gs-coupled β-adrenergic receptors have been implicated in glycogenolysis and ANLS-dependent learning. Pharmacological activation of β_2_-adrenergic receptors in vivo enhanced learning in young chickens, and this effect was blocked by inhibiting glycogenolysis [72]. In rats, knockdown of astrocytic β_2_ receptors in the hippocampus impairs long-term memory formation after passive avoidance learning, and this phenomenon in reversed by injection of lactate into the hippocampus [26]. In addition to neurotransmitters, the ANLS is also activated by elevated extracellular K^+^, which can result from neuronal activity and excitotoxicity. In this case, lactate release from astrocytes is dependent on sAC activation and an intracellular cAMP increase [39]. Therefore, cAMP, which is generated downstream of GPCRs and sAC, can be an important regulator of the ANLS in astrocytes.

Lactate taken up by neurons has been shown to be important for neuronal excitability and plasticity. Intracellular application of an LDH inhibitor in astrocytes hyperpolarizes the resting membrane potential of hippocampal pyramidal cells [68]. In addition, extracellular application of LDH inhibitors or MCT inhibitors reduces the amplitude of excitatory postsynaptic currents (Figure 1) [73]. Lactate release after glycogenolysis is also crucial for long-term potentiation, which supports the idea that glycogenolysis and the ANLS are important for learning and memory. Lactate has been shown to be essential for the phosphorylation of learning-related proteins, including the transcriptional factor cAMP response element-binding protein (CREB) and cofilin, which reorganizes actin filaments [3]. In addition, plasticity-related immediate early genes such as *Arc*, *c-Fos*, and *Zif268* are also expressed after extracellular lactate application both in vitro and in vivo through a mechanism involving MCT-dependent neuronal NMDA receptor activation and intracellular Ca^2+^ increase (Figure 1) [74]. In addition to these physiological roles, lactate can be an emergency energy source and exert protective effects in conditions accompanied by energy deprivation and excitotoxicity, such as hypoglycemia and brain trauma [39,75]. On the other hand, the disruption of the ANLS can be a therapeutic target when neuronal excitability and plastic changes are undesirable. For example, LDH inhibition in a mouse model of epilepsy blocked seizures caused by excessive neuronal activity [68]. As for plastic changes, inhibition of the ANLS in the basolateral amygdala disrupts drug-related memory (e.g., cocaine), preventing drug-seeking behavior and relapse [66,76]. It has also been shown that inhibition of the ANLS in the spinal cord rescues long-term mechanical allodynia caused by drug-induced plastic changes [77].

### 3.3. Other Targets of Lactate

Recently, the lactate receptor G-protein-coupled receptor 81 (GPR81, also known as hydroxycarboxylic acid receptor 1 (HCA1 or HCAR1)) was found in astrocytic end-feet [78]. GPR81 is coupled to Gi and reduces the intracellular cAMP levels when activated [78]. In contrast, it has been shown that lactate or GPR81 agonists activate AC and increase cAMP levels in astrocytes (which results in the production of lactate), surprisingly, in a GPR81-independent manner (Figure 1) [42].

Lactate released from astrocytes in the locus coeruleus activates nearby noradrenergic neurons and increases noradrenaline release in a PKA-dependent manner, which does not require lactate uptake by neurons (Figure 1) [79]. Since noradrenaline can trigger glycogenolysis and lactate release from astrocytes, this study suggests the existence of positive feedback loops for lactate release in the brain.

In conclusion, astrocytic cAMP can regulate glycogenolysis and lactate release, which are the fundamental functions of astrocytes and the principal mechanisms of brain energy metabolism.

## 4. Astrocytes and Extracellular Maintenance

### 4.1. Astrocytic cAMP and Extracellular K^+^ Clearance

K^+^ is constantly released into the extracellular space by neuronal activity. Since [K^+^]_out_ directly affects the resting membrane potential of neurons, it is important to remove extracellular K^+^ and maintain [K^+^]_out_ homeostasis. Elevated [K^+^]_out_ can cause neuronal hyperexcitability and seizures, which can be life-threatening conditions [6]. Astrocytes are crucial in cleaning up and buffering extracellular K^+^, mainly through reuptake by the Na^+^/K^+^ ATPase and NKCC1 (Na-K-Cl cotransporter 1) and redistribution through Kir channels (inward rectifier potassium channels) and gap junctions (K^+^ buffering) [4].

The Na^+^/K^+^ ATPase is one of the major transporters for K^+^ clearance in neurons and astrocytes, extruding intracellular Na^+^ and importing K^+^ using ATP. The astrocytic Na^+^/K^+^ ATPase has high capacity and low affinity compared to the neuronal Na^+^/K^+^ ATPase; the former enzyme rapidly removes extracellular K^+^ when [K^+^]_out_ is high but does not function when [K^+^]_out_ is low. This functionality suggests that the astrocytic Na^+^/K^+^ ATPase is a potent K^+^ remover immediately after neuronal activity, when [K^+^]_out_ is transiently elevated [80]. Since the Na^+^/K^+^ ATPase requires energy to function, its activity is tightly linked to glycogenolysis. In fact, Na^+^/K^+^ ATPase-mediated K^+^ uptake is completely abolished by the blockade of glycogenolysis (Figure 1) [61]. Thus, increased astrocytic cAMP induced by neuromodulators or elevated [K^+^]_out_ may facilitate K^+^ uptake by the Na^+^/K^+^ ATPase through glycogenolysis.

**Figure 2:**
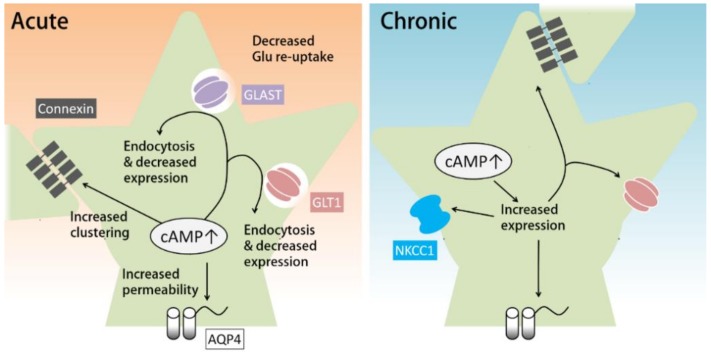
The role of cAMP in the maintenance of extracellular environment by astrocytes. (**Left**) Acute cAMP increase by cAMP analogues or adenylyl cyclase activators has been shown to decrease glutamate re-uptake through endocytosis of glutamate transporters. Astrocytic cAMP can also decrease the expression of glutamate transporters. In addition, short-term cAMP increase in astrocytes facilitates the clustering and also elevates water permeability of aquaporin 4 (AQP4). (**Right**) Long-term cAMP increase can induce the expression of AQP4, GLT1, connexin, and NKCC1.

Another transporter for the clearance of extracellular K^+^ is NKCC1, which functions especially rapidly when [K^+^]_out_ is greatly elevated. Similar to the Na^+^/K^+^ ATPase, NKCC1, which transports Na^+^ and Cl^−^ along with K^+^, also requires glycogenolysis to function (Figure 1) [61]. Notably, prolonged dbcAMP treatment (seven days) has been shown to increase the expression of NKCC1 (Figure 2, right) [81].

K^+^ buffering is a different mechanism by which astrocytes maintain [K^+^]_out_. Simply put, astrocytes import K^+^ from where [K^+^]_out_ is high and release K^+^ to where [K^+^]_out_ is low, both through Kir channels. However, a single astrocyte cannot redistribute K^+^ far away from where neuronal activity is occurring. Astrocytes are physically and electrically connected via gap junctions. The astrocytic network covers a larger area of the brain and redistributes K^+^ more efficiently than any single astrocyte could [4]. K^+^ buffering is important to prevent neuronal hyperexcitability induced by high [K^+^]_out_. Mutation of Kir4.1, which is a major Kir channel subunit in astrocytes, has been linked to epilepsy in humans [82]. In addition, the disruption of astrocytic gap junctions in mice has been shown to increase the frequency of seizure activity [83]. On the other hand, K^+^ redistribution mediated by astrocytic K^+^ buffering has been linked to the propagation of neuronal activity, which is important for neuronal oscillations but can also worsen seizures [83].

cAMP has been shown to regulate the function of gap junctions (Figure 2). The major components of astrocytic gap junctions are Cx30 (connexin 30) and Cx43 (connexin 43). cAMP can regulate connexin expression and gap junction assembly. A β-adrenergic agonist has been shown to upregulate the expression of Cx43 in glioma cell lines by activating Epac [84,85]. Corticotropin-releasing hormones can increase Cx43 expression and gap junction communication in cultured astrocytes and organotypic hippocampal slice cultures via the PKA pathway [86]. As for gap junction assembly, the PKA activator 8-Br-cAMP has been shown to increase gap junction assembly acutely in fibroblasts in a PKA- and Cx43-dependent manner [87].

### 4.2. cAMP Regulates Glutamate Reuptake by Astrocytes

Glutamate, as the major excitatory neurotransmitter in the brain, is released from excitatory neuronal terminals upon firing. Thus, rapidly removing glutamate from the extracellular space is essential for the maintenance of normal brain activity. Glutamate is mainly recycled by astrocytes via glutamate transporter 1 (GLT-1, also known as excitatory amino acid transporter 2, EAAT2) and glutamate aspartate transporter (GLAST, also known as EAAT1) [5]. GLT-1 and GLAST are expressed almost exclusively by astrocytes, with GLT-1 mainly in the forebrain and GLAST mainly in the cerebellum [5,88]. GLT-1, which accounts for 1% of total tissue protein in the forebrain, is one of the most highly expressed proteins in the brain. Similarly, GLAST is highly expressed in the cerebellum [89]. These expression patterns imply the importance of glutamate recycling by astrocytes. Recently, it has been found that GLT-1 swiftly removes extracellular glutamate through its high-affinity binding to glutamate and relocation on the astrocytic membrane [90]. Glutamate homeostasis by astrocytes is critical for brain health, as its disruption has been linked to several brain disorders including epilepsy, multiple sclerosis, and major depressive disorder [6,8,10].

Astrocytic cAMP has been shown to affect the expression, trafficking, and function of glutamate transporters (Figure 2). Prolonged treatment with cAMP analogues can increase the mRNA and protein expression levels of GLT-1 in cultured astrocytes [91,92,93]. In support of these in vitro studies, downregulation of GLT-1 in a rat model of depression was reversed by a PDE4 inhibitor via a PKA/CREB-dependent pathway [94]. However, other studies suggest that increased cAMP in astrocytes attenuates glutamate reuptake. For example, acute activation of A2A receptors inhibits glutamate uptake by GLT-1 [95,96]. Acute PKA inhibition in cortical glial culture has been consistently found to transiently increase glutamate uptake by GLAST [97]. In addition, hours of A2A receptor agonist treatment reduces the expression and protein levels of GLT-1 and GLAST via the PKA pathway [96], whereas astrocytic knockout of A2A receptors results in increased glutamate uptake and GLT-1 protein [98]. Accordingly, activation of the cAMP-PKA pathway induced by methamphetamine, anesthetics, or chronic morphine has also been shown to result in reduced glutamate uptake [99,100,101,102]. On the other hand, 24 h treatment with dbcAMP has been reported to increase the expression of GLT-1 and GLAST in retinal glial cells [103].

In summary, prolonged activation of the cAMP pathway in astrocytes increases the expression of glutamate transporters, while acute cAMP increases may downregulate glutamate uptake by astrocytes (Figure 2). One mechanism for this acute downregulation is the cAMP-induced endocytosis of glutamate transporters. Acute blockade of AC or downregulation of intracellular cAMP has been shown to increase glutamate uptake by inhibiting endocytosis of GLT-1 and GLAST [104]. An acute increase in cAMP has also been shown to reduce GLT-1 and GLAST expression; however, in this case, the effect does not last longer than 24 h [96].

In addition to transporters for glutamate recycling, xCT is another glutamate transporter expressed mainly by astrocytes; this protein takes up cysteine and releases glutamate in its place [105]. xCT is important for glutathione synthesis via cysteine uptake and has recently been found to be downregulated in a mouse model of depression; evidence also indicates that xCT is important for stress resilience [106]. Prolonged treatment with dbcAMP in astrocyte cultures has been shown to increase the expression of xCT [107,108]

### 4.3. Astrocytic cAMP and Water Transport

Water transport across the cell membrane is important for osmotic homeostasis and can affect cell volume and extracellular volume. In the brain, water transport mainly depends on the water channel aquaporin 4 (AQP4), which is expressed in astrocytes (as well as ependymocytes, which are responsible for the production of cerebrospinal fluid). Water flux usually occurs passively to adjust to osmotic changes; however, this process can cause astrocyte shrinkage or swelling, which leads to changes in extracellular volume and brain volume [109].

Brain volume can change under physiological conditions. For example, environment enrichment has been shown to cause an increase in hippocampal volume, which can be blocked by knockdown of AQP4 [110]. In addition, intensive language learning in humans can increase the volume of gray matter in the left inferoposterior temporal cortex, which is important for language skills. Interestingly, gray matter volume increase is related to language performance after intensive learning and is decreased in participants with a variant of the *AQP4* gene which causes low expression of AQP4 [110]. This result is consistent with findings showing that AQP4 deficiency can cause abnormalities in synaptic plasticity and spatial learning [111,112]. However, it should be noted that AQP4 Knockout (KO) not only impairs water flux but also impacts glutamate and K+ buffering [9]. For example, AQP4 KO mice exhibit slowed kinetics of [K^+^]_out_ change and increased seizure duration [113].

Pathological conditions such as brain injury or ischemic stroke can also cause an increase in brain volume, which is known as brain edema [114]. Brain edema caused by the swelling of cells (mainly astrocytes) can lead to high intracranial pressure, cerebral herniation, and death; however, brain edema has no established medical treatment [9,115,116]. AQP4, which governs water flux, obviously plays an important role in brain edema, as it has been shown that brain edemas induced by water intoxication or ischemic stroke may be reduced in AQP4 null mice [117,118].

The expression and function of AQP4 are subject to regulation by the cAMP pathway (Figure 2). Prolonged dbcAMP treatment has been shown to increase the expression of AQP4 in cultured astrocytes [91]. AQP4 can also be phosphorylated by PKA. Elevated [K^+^]_out_ induces an increase in intracellular cAMP (probably through sAC) and increases the permeability of AQP4 via PKA-dependent phosphorylation of AQP4. These conditions also cause an increase in astrocyte swelling caused by hypotonic stimulation [119].

NKCC1, whose activation is partly mediated by cAMP as previously described, is also a critical participant in brain edema because it imports not only water but also large quantities of ions, which can make the intracellular environment hypertonic and drive further water influx. It has been shown that NKCC1 deficiency can block astrocyte swelling induced by high [K^+^]_out_ [120]. NKCC1 has also been shown to mediate astrocyte swelling induced by ammonia [121].

Several studies have shown that activation of the cAMP pathway can affect astrocyte swelling and edema development. Isoproterenol has been shown to reverse hypotonically-induced astrocyte swelling in cultured cortical astrocytes. It has also been shown that hypotonic stimulation causes a decrease in the response of cAMP to adrenaline [116], which indicates that silencing of the cAMP pathway might be important in the causation of cell swelling. It has also been reported that activation of the β-adrenergic receptor, which results in increased cAMP levels, reduces hypotonically-induced intracellular Ca^2+^ increases that can contribute to astrocyte swelling [116,122]. In addition, it has been shown that increasing cAMP in retinal slices can block the swelling of Müller cells and intracellular cAMP increase in cultured Müller is important for the release of taurine, which mediates regulatory volume decrease (a response to cell swelling) [123,124]. On the other hand, in vivo studies have rendered different results. Administration of selective β_1_-adrenergic antagonists before or after ischemic stroke has been shown to attenuate edema development in rats, while selective β_2_ antagonists have no effect [125]. This phenomenon may be caused by cAMP regulation of NKCC1, as β_1_-receptor activation by isoproterenol activates glycogenesis, the Na^+^/K^+^ ATPase, and NKCC1 to accelerate regulatory volume increase (a response to cell shrinkage) of astrocytes induced by hypertonic stimulation [126]. However, it should be noted that in vivo administration of β_1_-adrenergic antagonists blocks neuronal β_1_-adrenergic receptors as well. This may explain the discrepancy between the results from in vitro and in vivo studies mentioned above.

In conclusion, in cultured astrocytes, prolonged cAMP pathway activation can induce the expression of GLT-1, NKCC1, connexin, and AQP4, which support the maintenance of extracellular homeostasis. On the other hand, acute cAMP pathway activation can up- or down-regulate this maintenance function through phosphorylation. Thus, the timecourse of cAMP increases in astrocytes can be crucial for the maintenance of the extracellular environment.

## 5. Immune Response and Astrocytes

### 5.1. Astrocytic cAMP Regulates Activation of NF-κB

Prolonged and excessive neuroinflammation is associated with numerous neurodegenerative diseases and mood disorders, including Alzheimer’s disease, multiple sclerosis, epilepsy, brain/nerve injury, and major depressive disorder [6,7,8,127,128]. Astrocytes are known to be active players in the immune response and neuroinflammation.

Nuclear factor kappa-light-chain-enhancer of activated B cells (NF-κB) is the master transcription factor in any inflammatory response. Upon activation, NF-κB translocates to the nucleus and increases the expression of cytokines and inflammatory factors. NF-κB activation in astrocytes has been shown to be detrimental in several diseases and disorders [127]. For example, genetic inactivation of astrocytic NF-κB in vivo leads to improved functional outcomes in a mouse experimental autoimmune encephalomyelitis (EAE) model, an experimental model of multiple sclerosis, decreasing immune cell infiltration into the brain and reducing inflammation-related gene expression [129]. In rodent models of Alzheimer’s disease, amyloid-β has been shown to activate NF-κB in astrocytes that express complement 3 (C3). C3 then compromises neuronal morphology and induces microglia-dependent synapse loss [130,131]. Astrocytic inactivation of NF-κB also ameliorates axon loss and preserves memory function in a mouse model of vascular dementia [132]. Recently, menin, a protein whose mutation is related to major depression disorder in humans, has been found to be downregulated in a mouse model of chronic stress. Conditional knockout of menin in astrocytes has been shown to activate NF-κB in astrocytes and is crucial for the expression of depression-like behavior [133].

**Figure 3:**
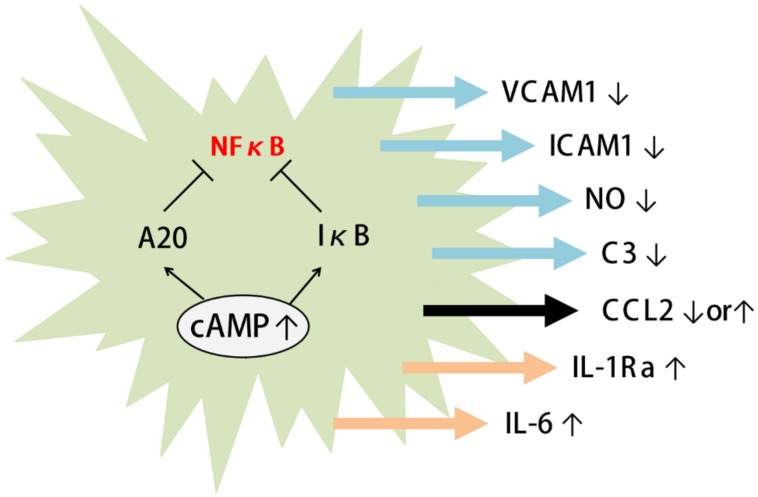
cAMP regulates immune response of astrocytes. The increase in astrocytic cAMP induces A20 and IкB, both of which suppress the activation of NF-кB. cAMP increase can reduce the expression of vascular cell adhesion molecule 1 (VCAM-1), ICAM-1, NO, and C3. cAMP increase has also been shown to either increase of decrease the expression of chemokine (C-C motif) ligand 2 (CCL2). In combination with TNFα, astrocytic cAMP increase can result in the increased expression of IL-1Ra and IL-6. Arrows indicate activating pathways. T-bars indicate inhibiting pathways.

A few studies have shown that astrocytic cAMP can suppress NF-κB activation (Figure 3). Noradrenaline and isoproterenol have been shown to induce IκB (nuclear factor kappa-light-chain-enhancer of activated B cells inhibitor) expression via the adrenergic β receptor. Since IκB binds to NF-κB and prevents its translocation to the nucleus, astrocytic cAMP may prevent NF-κB from being activated [134]. β-Receptor agonists, whether alone or in combination with TNFα (tumor necrosis factor alpha), can also induce the expression of A20 in cultured astrocytes [135]. A20 is an inhibitory factor of NF-κB that prevents NF-κB activation through multiple pathways [136]. Loss of A20 can cause gliosis in both astrocytes and microglia and can elicit spontaneous neuroinflammation [137,138]. Conditional knockout of A20 in astrocytes also results in intensified EAE symptoms accompanied by increased demyelination and peripheral immune cell infiltration [139].

Recently, it has been reported that microbial metabolites and type I interferons activate the aryl hydrocarbon receptor (AHR), which is highly expressed in astrocytes. The AHR exerts anti-inflammatory effects in a mouse model of EAE, while astrocyte-specific knockout of the AHR results in increased neuroinflammation and exacerbated disease [140]. These actions of AHR may be caused by its competitive binding to NF-κB subunit p65, in which it suppresses NF-κB activation [141]. AHR has also been shown to be activated by intracellular cAMP increases in a mouse hepatoma cell line, but whether this pathway exists in astrocytes is unknown [142].

Proinflammatory cytokines are known to lower astrocytic cAMP levels. A mixture of interleukin 1-β (IL-1β), TNFα, and IFN-γ (interferon gamma) can increase the activity of PDEs and induce PDE4B expression in cultured astrocytes. In the same study, astrocytes with elevated cAMP levels exhibited resistance to cytokine-induced inflammation [49]. Thus, cAMP in astrocytes may be a key molecule in suppressing temporary NF-κB activation and preventing prolonged NF-κB activation and subsequent neuroinflammation.

### 5.2. cAMP Pathways Regulate the Release of Cytokines and Inflammatory Factors

The cAMP pathway has been shown to regulate the expression of numerous cytokines and inflammatory factors by astrocytes. Acute application of isoproterenol to cultured astrocytes has been shown to decrease the expression of C3 and C-C motif ligand 5 (CCL5) induced by TNFα (Figure 3) [135]. Eight-day treatment with the cAMP analogue 8-Br-cAMP has been shown to decrease the expression of TNFα in cultured astrocytes [91]. Since TNFα is a classic proinflammatory cytokine and C3 is involved in microglia-dependent synapse elimination, astrocytic cAMP may provide a protective effect against neuroinflammation.

Isoproterenol also increases the expression of interleukin 6 (IL-6) and interleukin 1 receptor antagonist (IL-1Ra) when given in combination with TNFα (Figure 3) [135,143]. Another study has shown that noradrenaline can induce the expression of IL-1Ra as well as the decoy receptor of interleukin 1-β (IL-1β), known as interleukin 1 type II receptor (IL-1RII), in mixed glial cell culture via β-adrenergic receptors and the PKA pathway [144].

IL-1Ra and IL-1RII are both endogenous blockers of IL-1β. IL-1β has been shown to be detrimental in most brain diseases associated with neuroinflammation and is a key molecule that acts alongside TNFα to transform astrocytes into a neurotoxic form [145]. Thus, IL-1Ra and IL-1RII may exert therapeutic effects against neuroinflammation. For example, IL-1Ra or the blockade of IL-1R has been shown to be beneficial in epilepsy. Intrahippocampal application of IL-1Ra (one injection before seizure induction) or astrocyte-specific IL-1Ra overexpression has been shown to have a potent anticonvulsant effect against drug-induced seizures [146]. Intraperitoneal IL-1Ra treatment has been shown to block lipopolysaccharide (LPS)-induced enhanced epileptogenesis in kindled rat pups [147]. Similarly, IL-1Ra treatment has been found to block pediatric traumatic brain injury (TBI)-induced seizure susceptibility [148], probably due to its anti-inflammatory effect in TBI [149]. Recently, astrocyte-derived IL-1 has been shown to cause dysfunction in adult neurogenesis and memory in a West Nile virus neuroinvasive disease mouse model and enhance fear learning in a rat model of posttraumatic stress disorder, both of which are rescued by IL-1R blockade (the latter study uses IL-1Ra [150,151].

Another cytokine released by astrocytes upon cAMP elevation is IL-6. IL-6 is a classic proinflammatory cytokine that is important for B cell maturation and is involved in fever. Interestingly, IL-6 is also important for astrocyte differentiation during development [152]. IL-6 activates the Gp130-coupled IL-6 receptor in astrocytes. The IL-6-Gp130 pathway can activate Stat3 (signal transducer and activator of transcription-3), which is important for gliosis and scar formation. Loss of Stat3 attenuates gliosis and scar formation, resulting in widespread macrophage infiltration and a compromised behavioral outcome in both spinal cord injury and a mouse EAE model [153,154]. Gp130 can also activate the extracellular signal-regulated kinases (ERK) pathway to promote astrocyte survival in the lesion sites of EAE mice, which prevents progressive demyelination [155]. The protective role of the IL-6-Gp130 pathway may explain why astrocytic overexpression of IL-6 can ameliorate cuprizone-induced demyelination [156].

In addition to cytokines, the expression of nitric oxide, a signature molecule released by neurotoxic astrocytes, has also been suggested to be regulated by cAMP (Figure 3). The expression of inducible nitric oxide synthase (iNOS) induced by lipopolysaccharide (LPS) or the combination of IL-1β and TNFα is reduced by activation of the cAMP/PKA/CREB pathway [157,158,159].

### 5.3. Astrocytes and Peripheral Immune Cell Infiltration

Astrocytes are an important source of the chemokine C-C motif ligand 2 (CCL2), which plays a crucial role in attracting peripheral immune cells into the brain. In EAE mice, massive CCL2 expression has been found in astrocytes. Furthermore, EAE mice in which CCL2 was conditionally knocked out in astrocytes have been found to have reduced macrophage accumulation and T cell infiltration in the white matter of the spinal cord, and these mice have exhibited reduced axonal loss in the late stage of EAE [160,161]. CCL2 expression by astrocytes has been shown to be regulated by cAMP. CCL2 expression is increased upon 24 hours of treatment with noradrenaline, isoproterenol, and dbcAMP in cultured astrocytes [162]. On the other hand, prolonged 8-Br-cAMP treatment (eight days) results in decreased CCL2 mRNA levels (Figure 3) [91]. It should be noted that other cell types can also release CCL2, and probably at an earlier stage than astrocytes. CCL2-C-C chemokine receptor type 2 (CCR2) signaling has been shown to be crucial for monocyte-induced neuronal death after status epilepticus. However, CCL2 expression has been detected mainly in microglia and neurons [163]. Recently, pericytes have been found to be an immediate source of CCL2 after LPS injection [164]. CCL2 expression and infiltration of peripheral cytokines also have also been seen to occur in a mouse social defeat stress model, but the source of CCL2 has not been identified in this case [165].

Astrocytes also express the adhesion molecules ICAM-1 and vascular cell adhesion molecule 1 (VCAM-1), which mediate the adhesion of lymphocytes and monocytes and may subsequently mediate the infiltration of peripheral immune cells. Indeed, ICAM-1 null mice have been shown to develop a reduced severity of EAE with limited T cell infiltration and proliferation, and astrocytic VCAM-1 has been shown to be essential for T cell infiltration in a mouse EAE model [166,167]. Proinflammatory cytokines such as TNFα and IL-1β can increase ICAM-1 and VCAM-1 expression in astrocytes. Interestingly, pharmacological elevation of cAMP signaling reduces the expression of ICAM-1 and VCAM-1 in astrocytes (Figure 3) [135,168]. These findings suggest that increased astrocytic cAMP may reduce the infiltration of peripheral immune cells by reducing adhesion.

Astrocytes are also potential antigen-presenting cells since they express class II major histocompatibility complex (MHC) and B7 costimulatory molecules in certain circumstances. 5-HT_4_ receptor and β-adrenergic receptor activation, which lead to an increase in cAMP, have been shown to inhibit IFN-γ-induced expression of MHC class II and B7 costimulatory molecules in cultured astrocytes [30,169]. Antigen presentation is particularly important in multiple sclerosis. Myelin debris has been found in astrocytes in EAE models and multiple sclerosis patients, and IFN-γ-treated astrocytes have been shown to activate T cells through antigen presentation; however, it has also been reported that T cell activation in an EAE mouse model does not require astrocytes [170]. Thus, even though astrocytic cAMP may regulate astrocyte-dependent antigen presentation, antigen presentation by astrocytes might normally be rare in vivo.

In conclusion, astrocytic cAMP exerts anti-inflammatory effects through multiple pathways. Given that inflamed astrocytes and immune cell infiltration have been linked to numerous brain diseases, elevating cAMP in astrocytes may provide a protective effect against destructive inflammation.

## 6. Neurotrophic Factors from Astrocytes

### 6.1. cAMP-Dependent Release of Trophic Factors from Astrocytes

Brain-derived neurotrophic factor (BDNF) is one of the most prevalent and studied neurotrophic factors in the mammalian brain; this molecule and other neurotrophins such as nerve growth factor (NGF), neurotrophin-3 (NT-3) and NT-4 are required for several processes that include cell morphogenesis, neural circuit formation, synaptic plasticity, and neuronal and glial health [171,172,173].

Neurotrophins are mainly expressed in neurons; however, many studies have shown that astrocytes can also be a source of neurotrophins. Monoamines including noradrenaline, serotonin, and dopamine are known to induce BDNF expression in both cortical and cerebellar astrocytes in vitro [174]. Noradrenaline administration has also been shown to cause upregulation of BDNF expression in cultured Müller cells, the predominant glial cells in the retina [175]. Serotonin can increase NGF expression in striatum astrocytes [176]. Noradrenaline and dopamine are also able to increase NT-3 production by astrocytes in vitro [177].

Monoamine-induced neurotrophin release from astrocytes is regulated by cAMP signals. Noradrenaline-induced BDNF expression in astrocytes is mainly dependent on the β_1_ and β_2_ receptors and partly dependent on the α1 receptor [178]. The β-adrenergic receptor agonist isoproterenol increases BDNF and NGF expression in rat cortical and striatal astrocyte cultures [179]. Dopamine also increases BDNF expression in a CREB-dependent manner through β-adrenergic receptors in cultured rat cortical astrocytes [33]. Noradrenaline- and dopamine-induced NT-3 expression is dependent on the cAMP/PKA pathway as well [177]. Serotonin, however, is not effective at increasing cAMP levels in neonatal cortical astrocytes but can do so in adult cortical astrocytes [176]. Direct activation of cAMP pathways in astrocytes using dbcAMP or forskolin can increase BDNF expression in vitro to the same extent that β-receptor agonists do [178].

### 6.2. Functions of Astrocyte-Derived Trophic Factors

Many studies have shown that astrocyte-derived neurotrophins are neuroprotective in several models of disease and injury. Astrocytic expression of neurotrophins is upregulated after injury [179,180]. In a rat sciatic nerve injury model, BDNF, NGF, and NT-3 expression levels have been found to increase in spinal astrocytes under the conditions of astrogliosis. Inhibition of astrogliosis with fluorocitrate decreases the expression of neurotrophins in astrocytes and delays both axonal regeneration and functional recovery in vivo, which suggests a protective role of astrocyte-derived neurotrophins against injury [180]. Cuprizone-induced demyelination induces astrocytic expression of BDNF, which may support myelin protein synthesis after lesions [181]. In primary astrocyte culture and brain slice culture models of Huntington’s disease, mutant huntingtin protein reduces astrocytic release of BDNF [182]. Culture medium from astrocytes treated with quetiapine, an antipsychotic drug that induces BDNF expression in astrocytes but not in neurons, protects cultured neurons from β-amyloid-induced neuronal toxicity in a BDNF/TrkB-dependent manner [183]. Similarly, astrocyte-derived BDNF attenuates neuronal death induced by the anesthetic propofol in primary cultures from neonatal rats [184]. In addition, astrocytic neurotrophins are important for glial health. BDNF activates the truncated BDNF receptor in astrocytes and increases astrocyte viability under serum deprivation [185].

The physiological functions of astrocytic neurotrophins are still obscure. An interesting mechanism has been proposed involving astrocytic BDNF release and memory. Astrocytes have been shown to take up neuronal pro-BDNF through the p75 receptor and probably release BDNF in response to neuronal activity to induce long-term potentiation (LTP) upon memory acquisition [186]. Another paper has recently shown that mice exposed to an enriched environment exhibit astrocytic hypertrophy along with enhanced pro-BDNF immunoreactivity in the hippocampus [187].

### 6.3. Astrocytic cAMP and BDNF in Depression

Numerous postmortem studies showing decreased astrocyte density in patients with major depressive disorder have placed astrocytes at the center of its pathogenesis [10]. Recently, upregulated expression of astrocytic Kir4.1 in the lateral habenula has been shown to be a direct cause of depression-like behavior in mice, which suggests that, in some cases, depression can be a gliopathy [188]. Therefore, astrocytes are considered one of the target cell types for antidepressants. Specifically, the role of astrocytic BDNF has been in focus. Lentivirus-driven astrocytic BDNF overexpression in the hippocampus has been shown to elicit anxiolytic effects and increase neurogenesis in the dentate gyrus, which is considered to have an antidepressant effect [189]. Another study has shown that a single bilateral infusion of BDNF into the hippocampus is sufficient to induce a relatively rapid and sustained antidepressant effect [190]. Multiple antidepressants including selective serotonin reuptake inhibitors (SSRIs), tricyclic antidepressants (TCAs), and ketamine all increase BDNF levels or require BDNF expression for their antidepressant action [191].

Many antidepressants have been shown to increase BDNF levels in astrocytes. The TCA amitriptyline increases the levels of BDNF mRNA in rat cortical and hippocampal astrocyte cultures but not in cortical neuronal cultures [192,193,194]. The SSRIs fluoxetine and paroxetine also upregulate BDNF mRNA expression in mouse cortical astrocytes [195]. The TCA imipramine and the SSRI escitalopram increase BDNF mRNA in rat cortical astrocyte cultures [196].

The cAMP/PKA pathway has been shown to be involved in antidepressant-induced BDNF expression in astrocytes. Chronic antidepressant treatment increases the phosphorylation of CREB and CRE-mediated gene transcription in the cortex, amygdala, and hippocampus [197]. Consistent with these findings, imipramine induces BDNF expression in cultured astrocytes by activating CREB via the PKA pathway [198]. Fluoxetine has also been shown to activate PKA in vitro [199]. Similarly, 1,3,7-trihydroxyxanthone, a compound isolated from an herbal medicine that was used to treat forgetfulness and depression in ancient China, increases the expression of NGF and BDNF in cultured rat cortical astrocytes in a cAMP- and ERK-dependent manner [200]. However, the mechanisms of cAMP elevation in astrocytes remain unexamined in these cases.

Chronic administration of fluoxetine exerts antidepressant effects through increased BDNF expression in hippocampal astrocytes in vivo. In this paper, fluoxetine and ATP induce BDNF expression in vitro through purinergic receptors, including P2Y11 and A2B receptors, in a cAMP/PKA-dependent manner [201]. Another study suggests that antidepressants including ketamine and escitalopram increase intracellular cAMP levels in astrocytes via relocation of the Gαs subunit of GPCRs from lipid raft domains to nonraft domains in the membrane [202,203,204]. Gαs subunits located in raft domains are unable to associate with AC [205], which indicates that relocation of the Gαs subunit to nonraft domains can increase the cAMP response. Indeed, ketamine treatment increases the cAMP elevation caused by isoproterenol in the C6 glioma cell line and facilitates cAMP accumulation when administered by itself. In turn, ketamine also increases phosphorylation of PKA-associated proteins and BDNF in primary astrocyte cultures [202].

It has also been shown that antidepressants induce astrocytic BDNF expression through their ability to block the potassium channel Kir4.1. Additionally, Kir4.1 knockdown induces BDNF expression in cultured cortical astrocytes [206]. However, whether the cAMP/PKA pathway is involved in this mechanism remains unknown.

However, astrocytic involvement in the action of antidepressants is not completely dependent on BDNF. Anomalies in astrocytic glutamate uptake, especially decreased expression of GLT-1, may also play a role in depression [10]. In addition, blockade of glutamate reuptake by astrocytes in the prefrontal cortex induces anhedonia, which is a core symptom of major depressive disorder [207]. Ketamine has been shown to increase GFAP expression by astrocytes in vivo, which can result in the increased membrane expression of glutamate transporters. Other antidepressants may also increase glutamate transporter expression via the cAMP/PKA pathway [92,94,208].

In conclusion, astrocytic cAMP signals are crucial for neurotrophin expression in astrocytes, which is important for neuronal survival and for recovery from brain disorders including depression.

## 7. Conclusions

cAMP is a major second messenger that regulates astrocytic functions. In this review, we have discussed the importance of cAMP in basic astrocytic functions. Since many astrocytic functions are essential for neural function and thus for brain homeostasis, astrocytic cAMP can play an important role in maintaining the health of the CNS, and its anomaly may trigger or worsen pathological conditions. Thus, it is important to elucidate how astrocytic cAMP affects brain function both in health and disease. Although proinflammatory cytokines are known to lower astrocytic cAMP levels in vitro, which implies astrocytic cAMP anomaly in inflammation, direct evidence that links astrocytic cAMP anomaly to disease is lacking. Liu et al [94] have shown that cAMP pathways are suppressed in the hippocampus in a rat model of depression. In addition, the loss of noradrenergic neurons in locus coeruleus, which may result in an overall decrease of the cAMP component, has been observed in Parkinson’s and Alzheimer’s disease [209]. However, astrocyte-specific cAMP anomaly has yet to be identified in disease.

cAMP imaging can provide direct evidence on whether and how astrocytic cAMP levels are changed under healthy and diseased conditions. Specifically, because astrocytes possess complex and polarized morphology, it is necessary to observe cAMP increases at a subcellular level to understand how this second messenger can modulate different astrocytic functions [16]. Additionally, the temporal dynamics of cAMP in astrocytes should be clarified because long-term and short-term cAMP increases can activate different signaling pathways in a cell [210,211,212]. Thus, it is important to understand the spatiotemporal features of astrocytic cAMP signaling. Fortunately, studies focusing on cAMP imaging are accumulating, and more efficient cAMP sensors are being developed [29,116,213,214].

Astrocyte-specific manipulation of cAMP is indispensable to clarify the role of astrocytic cAMP in brain functions. Photoactivated adenylyl cyclase (PAC), an optogenetic tool that can increase cAMP levels in response to blue light, can be useful for activating the cAMP pathway. PAC was first identified in *Euglena gracilis,* and several other types of PAC have been discovered in microbes since then [215,216,217]. PACs have demonstrated their utility in investigating the roles of cAMP signaling in neurite growth, sperm mobility and behavioral changes [212,218,219]. Recently, a photoswitchable AC that enables faster light-based deactivation of enzyme activity has also been reported [220]. However, methods to block the astrocytic cAMP pathway are still lacking.

With these new technical approaches including the proper imaging and manipulation of astrocytic cAMP signaling, the role of astrocytic cAMP in health and disease will be unveiled in the near future.
